# Complete Genome Sequence of Halomonas venusta Type Strain DSM 4743, a Moderately Halophilic Marine Bacterium

**DOI:** 10.1128/MRA.00144-21

**Published:** 2021-05-06

**Authors:** F. Martinez-Abarca, L. M. Hernández-Soto, H. C. Ramírez-Saad, J. F. Aguirre-Garrido

**Affiliations:** a Structure, Dynamics, and Function of Rhizobacterial Genomes, Department of Soil Microbiology and Symbiotic Systems, Estación Experimental del Zaidín-CSIC, Granada, Spain; b Doctorado en Ciencias Biológicas y de la Salud, Universidad Autónoma Metropolitana-Lerma, Lerma, Estado de México, Mexico; c Departamento Sistemas Biológicos, Universidad Autónoma Metropolitana-Xochimilco, Mexico City, Mexico; d Departamento de Ciencias Ambientales, Universidad Autónoma Metropolitana-Lerma, Lerma, Estado de México, Mexico; Georgia Institute of Technology

## Abstract

Here, we report the genome sequence of Halomonas venusta strain DSM 4743^T^, a moderately halophilic marine bacterium. This type species genome consists of a 4.3-Mb chromosome, with 3,777 protein-coding genes, 60 tRNA loci, and 6 complete rRNA operons, plus a 6.1-kb plasmid termed p4743-A.

## Abstract (precis)

Here, we report the genome sequence of Halomonas venusta strain DSM4743^T^, a moderately halophilic marine bacterium. This type species genome consists of a 4.3-Mb chromosome, with 3,777 protein-coding genes, 60 tRNA loci, and 6 complete rRNA operons, plus a 6.1-kb plasmid termed p4743-A.

## ANNOUNCEMENT

Type strains serve as important genome references in bacterial taxonomy and systematics, being essential resources for microbiology. The *International Journal of Systematic and Evolutionary Microbiology* recently announced a strong recommendation for new taxon descriptions to provide genome sequence data (1). Thus, a genomic “gold standard” is required for consistent microbial species definition (2). To achieve this, a fundamental step is to sequence the type strains of validly published prokaryotic species. Strain DSM 4743 (= ATCC 27125 = CCUG 16063 = LMG 3445 = CIP 103201 = JCM 20634 = NBRC 102221) is the type strain of the species Halomonas venusta (3–5), one of the currently validly published species of the genus *Halomonas* (6). *Halomonas* is the largest genus of the *Halomonadaceae* family, which was proposed by Franzmann et al. (4) to accommodate moderately halophilic and marine bacteria. Defined in part by their salt tolerance, strains of the genus *Halomonas* have promising applications in biotechnology as sources of enzymes, compatible solutes, biosurfactants, or exopolysaccharides, among other products. Moreover, they can be used as cell factories to produce recombinant proteins (7). To date, only four genomes are available for H. venusta species, although more than 200 genomes corresponding to the genus *Halomonas* are available in the NCBI database. Here, we announce the assembled genome sequence of the type strain of the H. venusta species, strain DSM 4743, which was isolated from marine water in Hawaii (5).

The strain obtained from the DSMZ was grown in complete LBS10 medium (8). Genomic DNA was purified with the RealPure genomic DNA extraction kit (Durviz S.L.). Sequencing was performed on an Illumina MiSeq platform, at the Genome Sequencing Unit of the CGEB-Integrated Microbiome Resource, Dalhousie University (Canada), using a shotgun paired-end library according to standard protocols. A total of 946,804 paired-end reads were obtained (average length, 233 nucleotides), predicting 40-fold coverage of the genome. The quality (Phred) score was 30 or higher for 80.9% of the bases, and these qualified reads were processed with Geneious Prime software and the Geneious *de novo* assembly tool (v.11.1.5), using default parameters (9). From these analyses, 18 contigs were selected according to a minimum of 20× coverage (*N*_50_, 356,337 bp; average length, 236,801 bp; longest contig, 837,500 bp). Contigs were joined and the genome was nearly closed by using the tools of the Geneious Basic platform (9), further considering detailed observations of relevant sequencing reads mapping at the borders of each contig; the genomes of Halomonas venusta MA-ZP17-13 (GenBank accession number CP034367) and *Halomonas* sp. strain GST (GenBank accession number NZ_CP020562) were used as references when required. The reported genome consists of 4.28 Mb distributed into two replicons, i.e., the chromosome (4,276,113 bp, with a GC content of 52.8%) and a small plasmid termed p4743-A (6,148 bp, with a GC content of 51.5%). Annotation was carried out with the NCBI Prokaryotic Genome Annotation Pipeline (PGAP) (v. 5.0) (10), which predicted 3,777 protein-coding genes, 60 tRNA loci, and 6 complete rRNA operons. Furthermore, a CRISPR array belonging to a class 1 CRISPR-Cas system, type 1F, was identified by using the CRISPRCasTyper platform (11).

This publicly available reference genome for Halomonas venusta provides significant support for the analysis of metagenomic data sets (8, 12), and it is crucial for understanding the species’ fundamental traits and facilitating further work on this species at the genomic level.

### Data availability.

The sequences of the H. venusta DSM 4743 chromosome and the small plasmid p4743-A have been deposited in GenBank under accession numbers CP066539 and CP066540, respectively. The raw data have been deposited under SRA accession number SRP305761.
